# Complete Genome Sequence of Streptococcus gallolyticus Strain XH2168

**DOI:** 10.1128/mra.00105-23

**Published:** 2023-03-22

**Authors:** Siyuan Yang, Yue Yao, Feng Zhao, Yunsong Yu, Xiaoting Hua

**Affiliations:** a Department of Infectious Diseases, Sir Run Run Shaw Hospital, Zhejiang University School of Medicine, Hangzhou, China; b Key Laboratory of Microbial Technology and Bioinformatics of Zhejiang Province, Hangzhou, China; c Regional Medical Center for National Institute of Respiratory Diseases, Sir Run Run Shaw Hospital, School of Medicine, Zhejiang University, Hangzhou, China; d Clinical Laboratory Department, Sir Run Shaw Hospital, Zhejiang University School of Medicine, Hangzhou, Zhejiang, China; e Key Laboratory of Precision Medicine in Diagnosis and Monitoring Research of Zhejiang Province, Hangzhou, Zhejiang, China; University of Rochester School of Medicine and Dentistry

## Abstract

We assembled a complete genome sequence of Streptococcus gallolyticus strain XH2168 by combining the sequencing results from both the Illumina and Oxford Nanopore platforms. The assembled genome comprises 2,392,629 bp, with 37.69% G+C content and 2,265 protein-coding genes.

## ANNOUNCEMENT

Streptococcus gallolyticus belongs to group D Streptococcus, and it is also a conditionally pathogenic bacterium. S. gallolyticus is one of the main causes of infective endocarditis, reportedly causing 2% to 10% of cases (1). In addition, it is one of the few intestinal bacteria that has been consistently linked to colorectal cancer (CRC) and spinal epidural abscess (2, 3). To contribute to clinical practice, an in-depth study of this bacterium is warranted. Nevertheless, to date, there are only 49 complete genome sequences related to S. gallolyticus in the National Center for Biotechnology Information (NCBI). Here, we report the complete genome sequence of S. gallolyticus XH2168, which will provide an S. gallolyticus reference sequence for future genomic research. Ethical approval was obtained from the Ethics Committee of Sir Run Run Shaw Hospital, Zhejiang University School of Medicine (approval number 20190109-1), and informed consent was obtained from the participant.

S. gallolyticus XH2168 was isolated from a patient’s blood at Sir Run Run Shaw Hospital in Hangzhou, China. First, a positive blood culture was incubated for 5 days at 35°C in oxygen with 10% carbon dioxide. Then, the bacterium isolated from the positive blood bottle was cultured on Columbia blood plates for 48 h in a 5% carbon dioxide incubator. Finally, a single colony was selected from the Columbia blood plate and identified by mass spectrometry. Genomic DNA was extracted using a QIAamp DNA minikit (Qiagen, Hilden, Germany). To ensure that the genomic DNA was not degraded, we used agarose gel electrophoresis to check the integrity. The high-quality genomic DNA was prepared for sequencing using both the Illumina and Oxford Nanopore Technologies (ONT) platforms. In addition, the isolate (XH2168) was reconfirmed as S. gallolyticus by 16S rRNA gene analysis prior to genome sequencing. A NEXTflex rapid DNA sequencing (DNA-Seq) kit was used to construct Illumina sequencing libraries; the native barcoding expansion set (EXP-NBD104) and SQK-LSK109 ligation sequencing kit were used to construct Nanopore sequencing libraries without size selection or shearing. Qubit v3.0 (Invitrogen, USA) was used to check the quality and quantity of the pooled libraries before sequencing.

The hybrid assembly was implemented using the filtered MinION raw reads for assembly using Raven v1.1.10 (4) with default parameters. Next, Trycycler (5) was used to confirm the circularity of the complete genome sequence, and errors were corrected with the Illumina reads using Pilon v1.24 (6). The HiSeq X Ten platform (Illumina Inc., USA) and the Nanopore MinION platform (Oxford Nanopore Technologies, UK) were selected for whole-genome sequencing. Guppy v5.1.2 (Oxford Nanopore Technologies) was used in default mode with the parameters “–flowcell FLO-MIN106 –kit SQK-LSK109 –barcode_kits ‘EXP-NBD104 EXP-NBD114’ and high-accuracy.” Trimmomatic v0.30 was used to trim the Illumina reads (7). Default parameters were applied for all software during the hybrid assembly unless otherwise specified. Finally, the assembled complete genome sequence was annotated using the National Center for Biotechnology Information (NCBI) Prokaryotic Genome Annotation Pipeline (PGAP) (http://www.ncbi.nlm.nih.gov/genome/annotation_prok/).

For Illumina sequencing, 8,782,752 paired-end reads with an average size of 149.5 bp were obtained, and the average depth of sequencing was 549×. For Nanopore sequencing, a total of 74,339 reads were obtained, featuring a mean length of 9,210.8 bp and an *N*_50_ value of 14,444 bp; the depth of sequencing was 286×. Antibiotic resistance genes (ARGs) were identified using ABRicate v0.8.13 (https://github.com/tseemann/abricate). There are six ARGs in S. gallolyticus XH2168, including *tet*(L), *tet*(W), *erm*(B), *ant*(6), *lun*(B) and *lsa*(E). The genomic features are summarized in Table 1. The addition of this new reference strain is important for future genomic analysis of S. gallolyticus.

**TABLE 1:** Genomic features of the Streptococcus gallolyticus XH2168 chromosome

Genomic feature	Data
Size (bp)	2,392,629
No. of genes (coding)	2,265
No. of CDSs^*a*^ (total)	2,302
With protein	2,265
Without protein	37
No. of tRNAs^*b*^	71
No. of ncRNAs	4
No. of rRNAs	6
No. of CRISPR arrays	2
%GC	37.69
GenBank accession no.	CP113954.2

a CDSs, coding DNA sequences.

b ncRNAs, noncoding RNAs.

### Data availability.

The whole-genome sequence of S. gallolyticus XH2168 has been deposited at GenBank under accession number CP113954.2. The BioSample and BioProject accession numbers are SAMN31395130 and PRJNA892746, respectively. The raw sequence data have been uploaded to the Sequence Read Archive (SRA) under accession numbers SRR22071521 (Nanopore) and SRR22071636 (Illumina).
